# Fruit and vegetables and cancer risk

**DOI:** 10.1038/sj.bjc.6606032

**Published:** 2010-11-30

**Authors:** T J Key

**Affiliations:** 1 Cancer Epidemiology Unit, Nuffield Department of Clinical Medicine, Oxford University, Richard Doll Building, Roosevelt Drive, Oxford OX3 7LF, UK

**Keywords:** fruit, vegetables, colorectal cancer, lung cancer, breast cancer

## Abstract

The possibility that fruit and vegetables may help to reduce the risk of cancer has been studied for over 30 years, but no protective effects have been firmly established. For cancers of the upper gastrointestinal tract, epidemiological studies have generally observed that people with a relatively high intake of fruit and vegetables have a moderately reduced risk, but these observations must be interpreted cautiously because of potential confounding by smoking and alcohol. For lung cancer, recent large prospective analyses with detailed adjustment for smoking have not shown a convincing association between fruit and vegetable intake and reduced risk. For other common cancers, including colorectal, breast and prostate cancer, epidemiological studies suggest little or no association between total fruit and vegetable consumption and risk. It is still possible that there are benefits to be identified: there could be benefits in populations with low average intakes of fruit and vegetables, such that those eating moderate amounts have a lower cancer risk than those eating very low amounts, and there could also be effects of particular nutrients in certain fruits and vegetables, as fruit and vegetables have very varied composition. Nutritional principles indicate that healthy diets should include at least moderate amounts of fruit and vegetables, but the available data suggest that general increases in fruit and vegetable intake would not have much effect on cancer rates, at least in well-nourished populations. Current advice in relation to diet and cancer should include the recommendation to consume adequate amounts of fruit and vegetables, but should put most emphasis on the well-established adverse effects of obesity and high alcohol intakes.

Interest in the possibility that fruit and vegetables might help to reduce the risk for various types of cancer dates back to at least 1975, when the results from a small prospective study suggested that, even after allowing for the effect of smoking, people with a low intake of vitamin A from foods such as carrots and milk were at increased risk for lung cancer (Bjelke, 1975). Around the same time, investigations of differences in cancer rates and diet between countries suggested that various dietary factors, including plant foods, might have important effects on cancer risk (Armstrong and Doll, 1975). Epidemiological research on fruit and vegetables and cancer then increased rapidly, and by 1992, a review of 156 studies concluded that ‘for most cancer sites, persons with low fruit and vegetable intake experience about twice the risk of cancer compared to those with a high intake, even after control for potentially confounding factors’ (Block *et al*, 1992). Furthermore, consideration of the potential biological effects of various constituents of fruits and vegetables suggested plausible mechanisms for protective effects, such as by reducing oxidative damage of DNA or increasing the activity of enzymes able to detoxify carcinogens (Steinmetz and Potter, 1991). This view was consolidated by an expert panel report published in 1997, which stated that there was ‘convincing’ evidence that high intakes of fruit and/or vegetables decrease the risk for cancers of the mouth and pharynx, oesophagus, stomach, colorectum and lung (World Cancer Research Fund/American Institute for Cancer Research, 1997). However, within another 10 years, an updated report coordinated by the same organisation downgraded these previous conclusions from ‘convincing’ to either ‘probable’ or ‘limited-suggestive’ (World Cancer Research Fund/American Institute for Cancer Research, 2007). The principal reason for this change in judgement was that the newer results from large prospective studies did not confirm the earlier results, which had come mostly from case–control studies.

This overview summarises the epidemiological evidence on the associations of total fruit and vegetable consumption with the risk for major cancer sites (gastrointestinal tract, lung, breast, prostate and overall cancer risk), concentrating on the results from large prospective studies or pooled analyses. The difficulties in investigating this topic and possibilities for further research are then discussed.

## Associations of fruit and vegetable intake with risk of major cancer sites

### Cancers of the oral cavity and pharynx

Cancers of the oral cavity and pharynx are caused mostly by tobacco and alcohol (IARC, 1990). The association of fruit and vegetables with risk has been investigated in several case–control studies, which have observed an ∼50% reduction in the risk of these cancers in people with high intakes of fruit and vegetables (IARC, 2003). Few prospective studies of these cancers have been published, but these have generally observed substantial reductions in risk; in a study in the United States of cancers of the oral cavity, pharynx and larynx, Freedman *et al* (2008a) reported that, compared with people who consumed ∼1.5 portions of fruit and vegetables each day, people with intakes of ∼5.8 portions per day had a relative risk of 0.71 (95% CI 0.55–0.92), and in a study in Europe of squamous cell cancers of the oral cavity, pharynx, larynx (and oesophagus), Boeing *et al* (2006) reported that people with a high intake of fruit and vegetables (∼7.7 portions per day) had a relative risk of 0.60 (95% CI 0.37–0.99) compared with those with a relatively low intake (∼2.5 portions per day). Thus, the observational data are consistent with the hypothesis that relatively high intakes of fruit and vegetables reduce the risk for these cancers, but this interpretation is based on a rather small number of cases (787 cases in Freedman *et al*, 2008a; 352 cases in Boeing *et al*, 2006). Furthermore, tobacco and alcohol cause large increases in the risk for these cancers and are usually associated with low intakes of fruit and vegetables (Serdula *et al*, 1996); therefore, although the statistical analyses of epidemiological studies adjust as carefully as possible for smoking and alcohol, it is possible that the observed associations with fruit and vegetables are really due to tobacco and alcohol – so-called ‘residual confounding’.

### Oesophageal cancer

There are two main types of oesophageal cancer: squamous cell carcinoma and adenocarcinoma. Smoking is a causal factor for both types, alcohol is an important risk factor for squamous cell carcinoma and obesity and gastro-oesophageal reflux are important risk factors for adenocarcinoma (Stewart and Kleihues, 2003). The possible role of fruit and vegetables has been investigated in several case–control studies, which on average have observed that people with relatively high intakes of fruit and vegetables have a 40–50% lower risk of total oesophageal cancer than people with low intakes of fruit and vegetables (IARC, 2003). The few prospective data available suggest that fruit and vegetables are inversely associated with the risk for squamous cell carcinoma, but not with the risk for adenocarcinoma (Freedman *et al*, 2007; Yamaji *et al*, 2008). Therefore, the data are consistent with the hypothesis that adequate intakes of fruit and vegetables reduce the risk for squamous cell carcinoma of the oesophagus, but it is still possible that the association seen is due to residual confounding by smoking and alcohol.

### Stomach cancer

The risk of stomach cancer is increased by chronic infection with *Helicobacter pylori*, and there is substantial evidence that risk is increased by high intakes of salt-preserved foods and salt (WHO, 2003). There is a longstanding hypothesis that risk may be reduced by adequate intakes of fruit and vegetables, perhaps due to their content of antioxidant nutrients such as vitamin C. Data from case–control studies support this hypothesis (IARC, 2003), but the results from prospective studies have mostly shown weak or null associations (IARC, 2003; World Cancer Research Fund/American Institute for Cancer Research, 2007; Freedman *et al*, 2008b). Overall, therefore, the evidence does not suggest that fruit and vegetables reduce the risk of stomach cancer, at least in relatively well-nourished populations.

### Colorectal cancer

Diet is thought to have a major role in the aetiology of colorectal cancer, but the relevant components of diet are still not well understood. The incidence of this cancer is generally high in populations with high intakes of meat and low intakes of staple plant foods (Armstrong and Doll, 1975), and it has long been suggested that dietary fibre might reduce risk (Burkitt, 1971). Fruit and vegetables are rich in dietary fibre and, although there are other important sources such as unrefined cereals, it might be expected that if dietary fibre reduces the risk of colorectal cancer, then a reduction in risk would be observed in association with high intakes of fruit and vegetables. However, although one large prospective study of dietary fibre and colorectal cancer risk has found an inverse association with risk (1721 cases, Bingham *et al*, 2005), the results of other large prospective studies have been less clear (8081 cases, Park *et al*, 2005; 2110 cases, Nomura *et al*, 2007; 2974 cases, Schatzkin *et al*, 2007), and large prospective studies have suggested that high intakes of fruit and vegetables have at the most a small inverse association with the risk for colorectal cancer (Table 1). In the Women's Health Initiative randomised trial, the intervention was a low-fat eating pattern aimed at reducing dietary fat intake and increasing consumption of vegetables, fruits and grains; there was an average increase in fruit and vegetable intake of 1.1 servings per day, and the dietary intervention did not cause any significant change in the incidence of colorectal cancer after 8 years (Beresford *et al*, 2006). Overall, the data do not show a clear association between fruit and vegetables and the risk for colorectal cancer, although they are compatible with a small reduction in risk.

### Lung cancer

Heavy smoking increases the risk of lung cancer by ∼50-fold, and smoking causes over 80% of lung cancers in Western countries (IARC, 1990; Peto *et al*, 2000). Many observational studies have found that lung cancer patients report a somewhat lower intake of fruits and vegetables than controls, but the effect of smoking is so large, compared with the small association with diet, that residual confounding by smoking is likely, and recent large prospective analyses with detailed adjustment for smoking have not shown a convincing association between fruit and vegetable intake and the risk for lung cancer (Table 1).

### Breast cancer

Much of the epidemiology of breast cancer can be explained by reproductive and hormonal factors; in relation to diet, the only factors definitely related to breast cancer risk are obesity in postmenopausal women and alcohol (Key *et al*, 2003). Several large prospective studies have investigated whether high intakes of fruit and vegetables might be associated with a reduced risk of breast cancer, but overall the results are close to null (Table 1 and Michels *et al*, 2007). In the Women's Health Initiative randomised trial, an increase in fruit and vegetable intake of 1.1 servings per day (combined with an increase in grain intake and a reduction in fat intake) did not cause a significant change in the incidence of breast cancer after 8 years (Prentice *et al*, 2006). It seems unlikely that high intakes of fruit and vegetables in general have a significant protective effect, but it is still possible that specific vegetables rich in isoflavones, especially soyabeans, might have a protective effect by reducing the oestrogenic stimulation of the breast cells (Michels *et al*, 2007).

### Prostate cancer

The aetiology of prostate cancer is not well understood. Risk is increased in men with relatively high plasma concentrations of insulin-like growth factor-I, and levels of this growth factor can be affected by diet, but more research on this pathway is needed (Roddam *et al*, 2008). In relation to fruit and vegetables, recent large prospective studies suggest that there is little or no association between total fruit and vegetable intake and prostate cancer risk (Kirsh *et al*, 2007). There has been much interest in the possibility that fruits and vegetables, such as tomatoes, which are rich in the carotenoid lycopene might reduce the risk for prostate cancer, but overall the data do not support this hypothesis (Kavanaugh *et al*, 2007). Studies of soyabeans and prostate cancer have suggested that this vegetable may help to reduce risk, but the results are not conclusive (Hwang *et al*, 2009).

### Overall cancer risk

Four large prospective studies have recently reported on the associations of fruit and vegetable consumption with overall cancer risk (Table 1). These results for all cancers provide some indication of potential public health impact, but need to be interpreted cautiously because they may be influenced by chance and confounding at cancer sites not thought to be linked to fruit and vegetable intake. There was no significant association of either fruit or vegetables with total cancer risk in the combined analysis of two Harvard cohorts (the Nurses' Health Study and the Health Professionals' Follow-up Study) or in the Japan Public Health Center-Based Prospective Study. In the EPIC study conducted in 10 European countries, there was a small but statistically significant reduction in total cancer risk associated with high consumption of both fruit and vegetables, which was largely due to associations with cancer sites caused by smoking and/or alcohol. In the NIH-AARP study, fruit was not significantly associated with cancer risk in men or women, and vegetables were not associated with risk in women; vegetables were inversely associated with cancer risk in men, but this association was not evident in an analysis restricted to never smokers. Together, these analyses of fruit and vegetables in relation to overall cancer risk indicate that there is little, if any, association except in relation to cancers caused by smoking and alcohol, and these associations might be due to residual confounding by these factors.

### Randomised trials of dietary antioxidants

Several large randomised trials have tested the hypothesis that supplements of *β*-carotene or other dietary antioxidants could prevent cancer. Apart from a possible beneficial effect of *β*-carotene combined with vitamin E and selenium in a poorly nourished population in China, no benefits have been found at any cancer site, and high-dose supplements of *β*-carotene may increase the risk of lung cancer in smokers (Bjelakovic *et al*, 2008; Druesne-Pecollo *et al*, 2010).

## Discussion

Table 2 summarises the observed associations of fruit and vegetable intake with the risk of common cancers. In the early 1990s, there was a widespread belief that an increase in fruit and vegetable consumption would produce important reductions in cancer rates; it now appears that this view was unduly optimistic, and it is useful to consider why research on this topic has been difficult to interpret and how to plan future research to clarify this area.

### Bias, confounding and measurement error in observational studies

Most of the early studies on fruit and vegetables and cancer risk were case–control studies. These studies can be affected by bias: the cases have been diagnosed with cancer and their report of ‘usual diet’ may be affected by their illness or treatment, and the controls are intended to be a random sample of the whole population under study, but typically the response rate among potential controls is well below 100% and those who respond are relatively ‘health conscious’. These two biases could explain the higher reported intake of fruit and vegetables among controls than cases in many case–control studies, and therefore more attention should be given to the results of prospective studies, in which comparisons within the study are not affected by these biases.

However, as with all observational studies, there remains the problem of confounding – the association of the factor under study with other factors which may be the true causes of the disease. Smoking and alcohol cause large increases in the risk for several cancers and are usually associated with low intakes of fruit and vegetables (Serdula *et al*, 1996); for example, in the NIH-AARP study, men and women with high intakes of fruit and vegetables were much less likely to smoke or to have a moderately high alcohol intake than men and women with low intakes of fruit and vegetables (Table 3). For lung cancer, heavy smoking increases risk by ∼50-fold (Peto *et al*, 2000), whereas high intakes of fruit may be associated with a decrease in risk by ∼10% (Table 1). Thus, the adverse effect of heavy smoking on the risk of lung cancer is of the order of 500 times larger than the putative beneficial effect of a high fruit intake, and the apparent association of fruit intake with lung cancer risk could easily be explained by small imperfections in the ability to adjust for smoking. Theoretically, the possible relationship of fruit with lung cancer might be clarified by studying people who have never smoked, but lung cancer in never smokers is a rare disease and the data accumulated so far from prospective studies are not sufficient to draw conclusions.

In the recent large prospective studies, there has been little evidence that fruit and vegetables are associated with the risk for most types of cancer studied. The few significant associations observed have been in cancers strongly associated with smoking and/or alcohol consumption. In the EPIC study, for example, the relative risk for a 200 g per day increment in fruit and vegetable consumption was 0.92 (95% CI 0.90–0.95) for cancers associated with smoking, but 0.98 (95% CI 0.97–1.00) for cancers not associated with smoking (Boffetta *et al*, 2010); it may be that fruit and vegetables ameliorate the adverse effects of smoking, but it is also possible that these results are due to confounding.

Questionnaires provide only moderately accurate estimates of food intake. Much of the measurement error is random, and the resulting misclassification of individuals tends to attenuate the size of any true associations of food intake with disease risk. The combination of confounding and measurement error makes it difficult to interpret the results of large studies when these show a small, but statistically significant, association of fruit and vegetables with cancer risk. Small associations might be explained by residual confounding, but on the other hand, if small observed associations are due to a real protective effect, then the true effect may be substantially larger and thus of greater public health importance.

### Topics for further research

It is unlikely that fruit and vegetables have a ‘broad spectrum’ protective effect against cancer, and likely that some of the associations observed for particular cancer sites are simply due to confounding, particularly by smoking. This conclusion implies that, at least in relatively well-nourished westernised populations, a general increase in total fruit and vegetable intake will not have a large impact on cancer rates. This overall conclusion does not imply that there are no beneficial effects to discover, but future progress will depend on better understanding of the mechanisms by which cancer develops.

Fruit and vegetables might reduce cancer risk by supplying nutrients and thus preventing nutrient deficiency. A certain level of intake is needed to prevent nutrient deficiencies, but intakes above that level do not make the relevant tissues ‘super healthy’ this could suggest that variation between individuals in fruit and vegetable intake would only affect cancer risk in populations in which some people had intakes so low that they were deficient in the relevant nutrients, and the importance of fruit and vegetables in populations, or subpopulations, with particularly low average intakes should be further investigated.

For most potential mechanisms by which fruit and vegetables might reduce cancer risk, some types of fruit and vegetables would be expected to be much more strongly associated with risk than others, and such associations might be missed in examinations of cancer risk in relation to total fruit and vegetable consumption. The best chance of identifying any true protective effects of fruit and vegetables may depend on better understanding of the aetiology of specific cancers. Much of the previous research on fruit and vegetables and cancer has been conducted without a good understanding of potential mechanisms. For example, many studies have focused on the hypothesis that carotenoids such as *β*-carotene might reduce cancer risk due to antioxidant effects, but the role of oxidation and antioxidants in the aetiology of cancer is not clear (Halliwell, 2007). There are, however, some cancers for which we now know quite a lot about the mechanism. For example, it is known that high levels of circulating oestrogens are associated with an increased risk of breast cancer in postmenopausal women; therefore, the possibility that isoflavones from soya beans might be protective is plausible if these chemicals can reduce the oestrogenic effect of endogenous hormones (Key *et al*, 2003). To test this hypothesis, studies need to have reliable estimates of long-term exposure to isoflavones, and to be large enough to detect moderate effects; the anti-oestrogen tamoxifen reduces breast cancer risk by ∼40% (Cuzick *et al*, 2003), and it is probably unrealistic to expect dietary isoflavones to have such a large effect.

## Conclusions

General nutritional principles indicate that healthy diets should include at least moderate amounts of fruit and vegetables, sufficient to prevent deficiencies of any nutrients, especially micronutrients such as vitamin C, which are mostly supplied by fruits and vegetables. However, the available data suggest that general increases in fruit and vegetable intake would not have much effect on cancer rates, at least in relatively well-nourished populations. Future research may be productive if it can be focused on biological pathways known to be relevant in the development of specific types of cancer, and can reliably assess long-term intakes of relevant fruits and vegetables. Currently, advice in relation to diet and cancer should include the recommendation to consume adequate amounts of fruit and vegetables, but should put more emphasis on the well-established adverse effects of obesity and high alcohol intakes on cancer risk.

## Figures and Tables

**Table 1:** Results from large prospective studies on fruit and vegetables and risk of colorectal cancer, lung cancer, breast cancer and all cancers

**Cancer site and study**	**Reference**	**Number of cancers**	**RR (95% CI) for high fruit consumption**	**RR (95% CI) for high vegetable consumption**	**RR (95% CI) for high fruit and vegetable consumption**
*Colorectal cancer*					
EPIC	van Duijnhoven *et al* (2009)	2819	0.88 (0.76–1.01)	0.92 (0.79–1.06)	0.86 (0.75–1.00)
NIH-AARP	Park *et al* (2007)	2048 Men	1.06 (0.91–1.23)	0.82 (0.71–0.94)	0.91 (0.78–1.05)
		924 Women	1.09 (0.88–1.36)	1.12 (0.90–1.38)	1.08 (0.86–1.35)
Pooling Project (colon only)	Koushik *et al* (2007)	5838	0.93 (0.85–1.02)	0.94 (0.86–1.02)	0.91 (0.82–1.01)
					
*Lung cancer*					
EPIC	Büchner *et al* (2010)	1830	0.80 (0.66–0.96)	0.96 (0.79–1.17)	—
NIH-AARP	Wright *et al* (2008)	3834 Men	0.91 (0.82–1.02)	0.93 (0.83–1.03)	0.93 (0.83–1.04)
		2201 Women	0.97 (0.84–1.11)	1.05 (0.92–1.21)	0.98 (0.85–1.13)
Pooling Project	Smith-Warner *et al* (2003)	3206	0.77 (0.67–0.87)	0.88 (0.78–1.00)	0.79 (0.69–0.90)
					
*Breast cancer*					
EPIC	van Gils *et al* (2005)	3659	1.09 (0.94–1.25)	0.98 (0.84–1.14)	—
Pooling Project	Smith-Warner *et al* (2001)	7377	0.93 (0.86–1.00)	0.96 (0.89–1.04)	0.93 (0.86–1.00)
					
*All cancers*					
EPIC	Boffetta *et al* (2010)	30 604	0.94 (0.90–0.98)	0.93 (0.89–0.97)	0.89 (0.85–0.93)
Japan Public Health Center-Based Prospective Study	Takachi *et al* (2008)	3230	1.02 (0.90–1.14)	0.94 (0.84–1.05)	0.96 (0.85–1.07)
NIH-AARP Diet and Health Study	George *et al* (2009)	35 071 Men	0.98 (0.95–1.02)	0.94 (0.91–0.97)	—
		15 792 Women	0.99 (0.94–1.05)	1.04 (0.98–1.09)	—
Nurses' Health Study and Health Professionals' Follow-up Study	Hung *et al* (2004)	9261	1.01 (0.95–1.06)	0.99 (0.95–1.04)	1.00 (0.95–1.05)

Abbreviations: EPIC=European Prospective Investigation into Cancer and Nutrition; NIH-AARP=National Institutes of Health-American Association of Retired Persons Diet and Health Study; Pooling Project=Pooling Project of Prospective Studies of Diet and Cancer, a pooled analysis of primary data from 14 (colon cancer) and 8 (lung cancer, breast cancer) prospective studies in North America and Europe, respectively; RR (95% CI)=relative risk (95% confidence interval) of cancer for individuals in the highest category of consumption compared with those in the lowest category.

**Table 2:** Summary: fruit and vegetables and risk of common cancers

**Cancer site**	**Current evidence for association with fruit and vegetables**	**Comments**
*Gastrointestinal tract*		
Oral cavity, pharynx, oesophagus	Consistent inverse association	Not clear if causal – might be due to residual confounding by other factors such as tobacco and alcohol
		
Stomach	Generally little or no association	—
Colorectum	Inconsistent, weak, inverse association	Could be due to protective effect of dietary fibre
Lung	Inconsistent, weak, inverse association	Could be due to residual confounding by smoking
Breast	Little or no association	—
Prostate	Little or no association	—

**Table 3:** Associations of fruit and vegetable intake with smoking and alcohol intake

	**Men**		**Women**	
**Fruit and vegetable intake**	**Current smokers (%)**	**Alcohol intake ⩾15 g per day (%)**	**Current smokers (%)**	**Alcohol intake ⩾15 g per day (%)**
*Fruit*				
Lowest fifth	21.8	38.3	26.6	17.2
Highest fifth	4.1	16.7	7.8	5.3
				
*Vegetables*				
Lowest fifth	16.9	32.1	20.6	12.6
Highest fifth	6.2	22.7	10.3	9.4

Data for 288 109 men and 195 229 women in the National Institutes of Health-American Association of Retired Persons Diet and Health Study, adapted from George *et al* (2009).
